# Breast Cancer Care in Ghana: A Review of the Continuum of Care and Progress Toward Universal Health Coverage

**DOI:** 10.1155/ijbc/3787700

**Published:** 2026-09-16

**Authors:** Shannon C. Lyden, Emma Gabriella O′Connor, Katie Kenny, Hannah Wang, Katerina L. Aris, Caroline Lommer, Jessica Keane, Ishmael Kyei, Anita Eseenam Agbeko, Abena Yeboah Aduse-Poku, Bridget Oppong, Andrea N. Riner

**Affiliations:** ^1^ The Ohio State University College of Medicine, Columbus, Ohio, USA, osu.edu; ^2^ School of Medical Sciences, Kwame Nkrumah University of Science and Technology, Kumasi, Ghana, knust.edu.gh; ^3^ Directorate of Oncology, Komfo Anokye Teaching Hospital, Kumasi, Ghana, kathhsp.org; ^4^ Division of Surgical Oncology, Department of Surgery, The Ohio State University Wexner Medical Center, Columbus, Ohio, USA, osu.edu

## Abstract

**Introduction:**

Breast cancer is the most prevalent cancer among women in Ghana, and improving outcomes requires coordinated progress across the continuum of care. This review synthesizes current evidence on breast cancer care in Ghana, including screening and diagnosis, treatment availability and utilization, survivorship, financial and transportation considerations, sociocultural determinants, and emerging innovations.

**Methods:**

A narrative literature review was conducted using iterative topic‐based searches across PubMed and Google Scholar, supplemented by searches on Google.com for Ghana‐based programs and on ClinicalTrials.gov for clinical trials. Ninety‐nine sources that were published in 2011–2026 and met relevance criteria were included. Secondary chi‐square and univariate logistic regression analyses of the 2022 Ghana Demographic and Health Survey examined associations between breast cancer screening and socioeconomic/geographic factors. Geographic heatmaps were created to illustrate regional variation in diagnostic and treatment service availability.

**Results:**

Screening and diagnostic imaging services are increasingly available but concentrated in urban centers, highlighting regional access opportunities. Pathology service shortages hinder timely diagnosis and subtyping, though ongoing investments in diagnostic infrastructure represent progress. Treatment services—including surgery, systemic therapy, and radiation—are available at select centers nationwide, and continued expansion should improve outcomes as earlier detection increases. Despite high national insurance enrollment, screening and treatment coverage gaps persist, underscoring the need for capacity building and policies to reduce financial burden and improve care continuity. Sociocultural beliefs and awareness shape health‐seeking behavior, making community‐based education key to early detection and treatment adherence. Ghana is growing capacity in emerging areas of breast cancer care, including genetic counseling and testing, fertility preservation, and clinical trial participation, which, although not widely available, may enhance patient outcomes.

**Conclusion:**

These findings highlight progress and opportunity in Ghana′s breast cancer care landscape, underscoring the value of investing in decentralized services, workforce development, capacity building, and patient‐centered strategies for equitable breast cancer care nationwide.

## 1. Introduction

Breast cancer poses an urgent and growing public health challenge in Ghana as the most commonly diagnosed cancer and the leading cause of cancer‐related death among Ghanaian women [1]. In 2020, breast cancer accounted for approximately 31.8% of all cancer cases in the country [2], diagnosed at an average age of only 49 years [3]. Epidemiologic data are limited by the absence of a national, population‐based cancer registry [4], which impairs the availability of high‐quality data necessary to monitor trends, allocate resources, and implement targeted interventions. Consequently, much of what is known about breast cancer in Ghana is derived from hospital‐based registries and local research, which may not fully capture the national landscape.

The current literature identifies factors that affect each aspect of care within the landscape of breast cancer care in Ghana. However, most of this literature consists of small‐scale studies at individual institutions or studies that focus solely on one aspect of breast cancer care. Thus, the literature lacks a synthesized resource that compiles verified information on available resources across all 16 regions of the country. This review is aimed at providing a comprehensive outline of the current landscape of breast cancer care in Ghana. By consolidating evidence across the breast cancer care continuum—from prevention to diagnosis, treatment, survivorship, and emerging innovations, this review seeks to illuminate both areas of progress and unmet needs. As the burden of breast cancer continues to rise, a thorough understanding of existing care frameworks is essential for designing sustainable, equitable, and contextually appropriate interventions.

## 2. Methods

**Figure 1:**
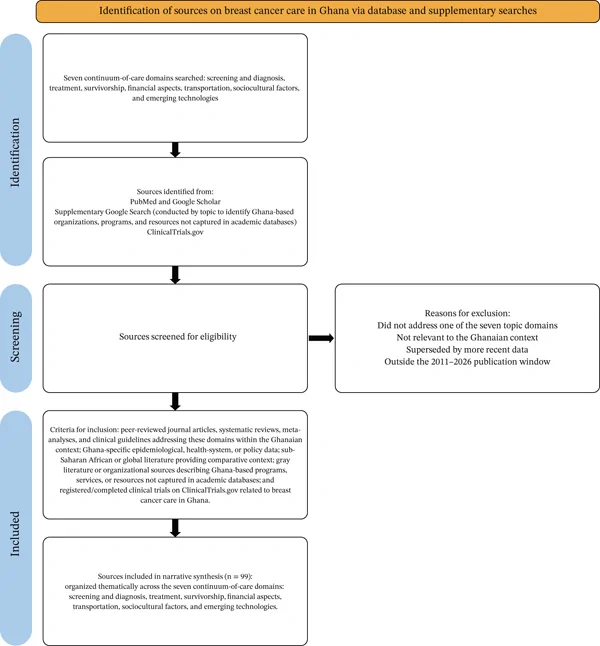
Literature search and selection process flow diagram, adapted from the PRISMA 2020 flow diagram for a narrative (nonsystematic) review [5].

A narrative literature review was conducted to characterize the current landscape of breast cancer care in Ghana across the continuum of care, including screening, diagnosis, treatment, survivorship, financial aspects, transportation, sociocultural factors, and emerging technologies. As this was a narrative (nonsystematic) literature review, searches were conducted iteratively by topic across PubMed and Google Scholar, rather than using a single fixed Boolean search string. Additionally, supplementary Google searches were conducted by topic to identify Ghana‐based organizations, programs, and resources not captured in academic databases. Between June 2025 and March 2026, the literature search was conducted by five co‐authors who were each assigned to a specific topic area to conduct the literature search. Searches were conducted by topic using free‐text search terms in both PubMed and Google Scholar, as well as peer‐reviewed journal articles. Search terms were tailored to each topic area, generally combining “breast cancer” and/or the relevant topic with “Ghana” (File S1). In addition, ClinicalTrials.gov was searched directly to identify relevant registered and completed clinical trials on breast cancer care in Ghana. Sources were included if they were peer‐reviewed journal articles, systematic reviews, meta‐analyses, or clinical guidelines addressing breast cancer across the seven continuum‐of‐care domains within the Ghanaian context; Ghana‐specific epidemiological, health system, or policy data; broader sub‐Saharan African or global literature providing comparative context for Ghana‐specific findings; gray literature or organizational sources describing Ghana‐based programs, services, or resources not captured in academic databases; or registered/completed clinical trials on ClinicalTrials.gov related to breast cancer care in Ghana. Sources were excluded if they did not address one of the seven topic domains, lacked relevance to the Ghana context, were superseded by more recent data, or fell outside the 2011–2026 publication window. Overall, 99 sources meeting relevance criteria were selected, with publication dates ranging from 2011 to 2026. Findings across all topic areas were subsequently reviewed by the two co‐first authors to ensure consistency and integration into a cohesive narrative synthesis organized thematically across the breast cancer care continuum (Figure 1).

Secondary analyses were conducted using data from the 2022 Ghana Demographic and Health Survey (GDHS), a nationally representative household survey employing a stratified, multistage cluster sampling design. Data were collected from approximately 18,000 households across all 16 regions of Ghana, including a specific women′s questionnaire given via interviews with over 15,000 women deemed of reproductive age, including ages 15–49, and a men′s specific questionnaire given to 7000 men deemed of reproductive age, including ages 15–59. GDHS data were used to examine patterns of health insurance coverage, health care utilization, out‐of‐pocket payments, and breast cancer screening–related indicators across socioeconomic and geographic strata [6]. The statistical software JMP was used to run a *χ* ^2^ test of significance between wealth quintiles, geographic status, and educational attainment by breast cancer screening status. Univariate logistic regression models were employed to calculate unadjusted odds ratios (ORs) for breast cancer screening by wealth quintile, education level, and residence, with each variable modeled independently. Overall significance for each variable was assessed using the likelihood ratio test. The software Datawrapper was utilized to create geographical heatmaps of the availability of breast cancer diagnostic and treatment services across the varying regions in Ghana [7].

## 3. Results

### 3.1. Screening and Diagnosis

#### 3.1.1. Clinical Breast Exam (CBE), Mammography, and Ultrasonography

**Figure 2:**
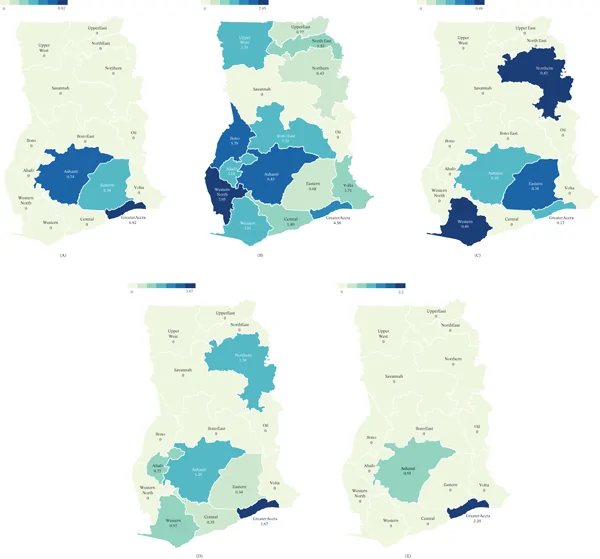
Heatmap of breast cancer services in Ghana. (A) Regional distribution of mammography units per one million people across Ghana. (B) Regional access to breast and axillary ultrasonography units per one million people across Ghana. (C) Regional access to on‐site breast cancer pathology services per one million people across Ghana. Slanted lines represent regions with excisional/incisional biopsy services available. (D) Regional distribution of computed tomography units per one million people across Ghana. (E) Regional distribution of MRI units per one million people across Ghana.

Screening for breast cancer worldwide includes a CBE, in which health care providers perform a physical exam of the breasts and draining lymph nodes, along with mammography [8], if resources and health care infrastructure allow. Among all modalities, mammography remains the gold standard in screening for breast cancer and reducing mortality by approximately 20% for women with average risk [9, 10]. Digital mammography has a sensitivity of 92% and a specificity of 96%, outperforming magnetic resonance imaging (MRI) (sensitivity = 68*%*, specificity = 95*%*) and breast ultrasound (sensitivity = 72*%*, specificity = 92*%*). Whereas these findings have been debated, with some studies showing more favor for the diagnostic power of breast MRI and breast ultrasound, the heightened ability of mammography to detect microcalcifications within the breast has continuously placed mammography at the highest priority when screening for breast cancer [11]. In Ghana, mammography imaging is available in 9 of the 16 regions (Figure 2A) [7]. Alongside mammography, ultrasound can be used to further evaluate dense tissues or distinguish solid masses from cysts [12]. Whereas ultrasonography alone is inferior to mammography, the addition of ultrasonography with mammography may improve the number of screening‐detected breast cancer cases [13]. Breast ultrasonography is available in either diagnostic centers or hospital settings in 14 of the 16 regions in Ghana (Figure 2B) [14]. One study estimates that 41.3% of the population lives within 1 h of mammography services and 69.5% of the population lives within 1 h of ultrasonography services [14].

Disparities in access to mammography parallel access to CBE. Using data extracted from a demographic survey of 2022, when comparing CBE screening across regions, those in the Oti Region had 44% lower odds, and those in the Upper East Region had 40% lower odds of receiving a CBE than women in the Western Region [15]. Several interesting factors associated with increased odds of receiving a CBE included listening to the radio at least once per week (AOR = 1.35, 95% CI: 1.20, 1.53), visiting a health facility within the past 12 months (AOR = 1.36, 95% CI: 1.23, 1.51), and prior screening for cervical cancer (AOR = 6.64, 95% CI: 5.51, 7.99) (Table 1) [15]. Health care training and education to expand CBE across regions provide an additional solution to increase earlier identification and diagnosis of breast cancer. Promising efforts are displayed regarding increased accuracy of CBE, as represented by the MammaCare computer training simulation, which, through implementation in various clinics in Ghana, has improved specificity and sensitivity in identifying breast cancer [16].

**Table 1:** Breast cancer screening rate and odds of screening in Ghanaian women aged 15–49 across demographic variables.

Demographic variable	% screened	Odds ratio (95% CI)	*p*value
Wealth quintile (*χ* ^2^ = 806.03, df = 4)			< 0.0001
Q1	7.7		
Q2	11.2		
Q3	15.4		
Q4	19.4		
Q5	33.8		
Q2 vs. Q1		1.51 (1.25–1.82)	< 0.0001
Q3 vs. Q1		2.18 (1.82–2.60)	< 0.0001
Q4 vs. Q1		2.88 (2.43–3.42)	< 0.0001
Q5 vs. Q1		6.11 (5.18–7.20)	< 0.0001
Q3 vs. Q2		1.44 (1.24–1.68)	< 0.0001
Q4 vs. Q2		1.91 (1.65–2.21)	< 0.0001
Q5 vs. Q2		4.05 (3.52–4.65)	< 0.0001
Q4 vs. Q3		1.32 (1.16–1.50)	< 0.0001
Q5 vs. Q3		2.81 (2.49–3.17)	< 0.0001
Q5 vs. Q4		2.12 (1.90–2.37)	< 0.0001
Education level (*χ* ^2^ = 907.22, df = 3)			< 0.0001
No education	9.1		
Primary	11.4		
Secondary	17.6		
More than secondary	47.3		
Primary vs. no education		1.29 (1.06–1.54)	0.0108
Secondary vs. no education		2.14 (1.84–2.48)	< 0.0001
More than secondary vs. no education		8.98 (7.57–10.66)	< 0.0001
Secondary vs. primary		1.66 (1.44–1.92)	< 0.0001
More than secondary vs. primary		6.98 (5.89–8.26)	< 0.0001
More than secondary vs. secondary		4.20 (3.75–4.71)	< 0.0001
Residence (*χ* ^2^ = 290.61, df = 1)			< 0.0001
Rural	12.3		
Urban	23.0		
Urban vs. rural		2.13 (1.95–2.33)	< 0.0001

*Note:* Source: Data derived from the 2022 Ghana Demographic and Health Survey (DHS), Ghana Statistical Service, ICF International. Screening rates reflect weighted estimates among women aged 15–49. Statistical significance (*p* < 0.0001) was determined using chi‐square tests for trend across wealth quintiles and education levels, and binary comparisons for urban versus rural residence. Odds ratios reflect univariate unadjusted logistic regression models estimated separately for each variable; wealth quintile, education level, and residence were not modeled simultaneously. *p* values reflect the overall effect likelihood ratio test for each variable.

#### 3.1.2. Pathology

Pathology is an essential component of breast cancer diagnosis, staging, and determination of appropriate systemic therapy. A nationwide assessment conducted between November 2020 and October 2021 found that 136 of 328 hospitals (41%) reported access to breast cancer pathology services, though only six had on‐site pathology laboratories [14]. Most hospitals relied on external referral systems, which have proven relatively effective, with approximately 75% of the population living within 1 h of a pathology‐capable facility. The national pathology workforce currently includes approximately 30 breast pathology specialists (one pathologist per 1,056,961 people), most of whom are concentrated in high‐volume centers, underscoring the need for expanded outreach to ensure equitable access in remote regions [14].

Fine needle aspiration (FNA) is widely used for initial tissue diagnosis, especially in regions with limited pathology infrastructure, and has demonstrated high sensitivity and positive predictive value when performed by skilled operators [17, 18]. Core needle biopsy (CNB) is increasingly available, particularly in major referral centers such as those in Greater Accra and the Ashanti regions (Kumasi), following targeted training programs [19]. CNB is preferred as tissue architectural analysis and immunohistochemistry (IHC) depend upon this technique, but its use is limited by the availability of equipment and trained personnel outside major urban centers [18, 19]. Surgical biopsies are more likely to be performed in tertiary hospitals, due to resource constraints in peripheral areas [20, 21]. Major urban centers have the highest concentration of pathology and surgical services, whereas peripheral regions (Savannah, Bono East, Oti, Ahafo, Western North, North East, Upper East, and Upper West) rely on referral pathways to centralized facilities for diagnostic confirmation [20–22]. Most hospitals outside these urban centers offer fragmented diagnostic services, with FNA being the most accessible technique (Figure 2C) [7, 17, 20, 22].

Ghana′s pathology services currently include three main diagnostic modalities: histopathology, IHC, and, to a very limited extent, molecular diagnostics. Histopathology is routinely used across most regional and tertiary hospitals, though processing is frequently centralized and subject to delays in tissue fixation and reporting. IHC serves as a critical step in tumor characterization to assess hormone receptor (HR) (estrogen [ER] and progesterone [PR]) status and human epidermal growth factor Receptor 2 (HER2) expression, allowing for subtype classification and systemic treatment planning. Of hospitals with pathology services that offer IHC on‐site, 38% conduct ER/PR testing, and 33% offer HER2 testing. Referral centers in Accra and Kumasi demonstrate over 80% consistency in performing these tests, enabling more standardized classification of tumors and appropriate treatment selection [20]. More advanced techniques, such as fluorescence in situ hybridization (FISH) and genomic profiling, remain uncommon and are largely confined to academic or pilot research settings due to cost, infrastructure, and workforce limitations. Within the breast cancer patient population diagnosed at Cape Coast Teaching Hospital (CCTH) between 2011 and 2019, 50% of patients were evaluated for HR status, and 47% were evaluated for HER2/neu overexpression. Forty‐seven percent of patients were evaluated for HER2 overexpression [23].

Practical determinants, such as turnaround time and insurance coverage, further shape whether available pathology services translate into timely, actionable diagnoses. In the same nationwide assessment, 94% of hospitals with breast cancer pathology access reported obtaining results within 1 month of specimen submission, but only 26% within 2 weeks, with the shortest turnaround time concentrated among hospitals using private laboratories (68% of hospitals) [20]. Although breast cancer services are formally included in the National Health Insurance Scheme (NHIS) benefits package [24], coverage is inconsistently realized in practice, and out‐of‐pocket payment for diagnostics, including pathology, is common. A single‐institution study at a regional hospital in the Eastern Region reported that pathology was not covered under the NHIS at the facility, with out‐of‐pocket charges for specimen processing and transport delaying diagnosis, and in some cases, led to specimens being discarded before analysis [25].

Encouraging progress is being made in building technical capacity. Ongoing investments in equipment, staff training, and quality assurance are helping to expand diagnostic reach. Efforts are also underway to strengthen workforce pipelines through postgraduate training and international partnerships. Diagnostic advancements are translating into more detailed knowledge of tumor biology in Ghana. A study from Korle Bu Teaching Hospital found that 49.4% of tumors were triple‐negative breast cancers (TNBCs) [26]. Proper diagnosis of tumor subtypes helps guide therapeutic decisions, especially since TNBC—more prevalent among younger Ghanaian women—is often more aggressive with larger tumor size and tends to have a higher oncological grade and lymph node metastasis [27]. Nationally, this higher proportion of aggressive subtypes compared with Western populations highlights the need for context‐specific treatment protocols. Pilot initiatives at centers such as Peace and Love Hospital are demonstrating that streamlined biopsy‐to‐IHC workflows and improved sample transport can reduce turnaround times and expand diagnostic reach [28]. With continued investment and decentralization, Ghana is on course to offer equitable, precise, and timely pathology services for breast cancer patients across all regions. Further, the University of Michigan (UM) is pursuing the global surgery initiative of an International Breast Cancer Registry through the UM–Komfo Anokye Teaching Hospital (KATH) partnership. This partnership has launched a training program in percutaneous core needle and punch biopsy as well as an annual refresher course to ensure updated training of faculty. The UM‐KATH partnership has also supported a pathology training program for KATH staff to develop an IHC program for on‐site testing [29]. Moreover, a comprehensive understanding of biopsy methods currently employed, as well as the histopathologic and IHC tools used to evaluate cancers, is essential for assessing diagnostic capacity and identifying gaps in service delivery.

#### 3.1.3. Staging and Advanced Imaging

Advanced imaging capacity for staging in Ghana is expanding but remains geographically concentrated and unevenly distributed. A 2021 national audit reported that 6 of 16 regions in Ghana lacked computed tomography (CT) scanners, leaving around 5 million people without ready access to CT imaging [30]. Eastern and Volta regions each had only one CT radiographer, and Bono, Bono East, Eastern, Volta, and Upper West each had only one radiologist at the time of the audit [30]. The Eastern Region had the lowest availability, with one scanner serving approximately 3,318,853 people (Figure 2D) [7, 30]. Across all facilities with CT scanners, 22% were nonfunctioning at the time of assessment, representing limitations with maintenance of costly equipment [30].

Access to MRI also displays regional disparities. A 2016 survey found that 67% of MRI units are in Greater Accra, leaving an estimated 40% of the population without regional access to MRI [31]. Further, overall availability was approximated at 0.5 MRI scanners per million population [31]. Bone scintigraphy is available at the National Radiotherapy Oncology and Nuclear Medicine Centre at the Korle Bu Teaching Hospital in the Greater Accra Region (Figure 2E) [7, 32]. Recent progress has been shown with the first PET‐CT machine now available at the Sweden Ghana Medical Centre as of May 2025 [33]. Further, the City Cancer Challenge (C/Can) has spearheaded the design of a nuclear medicine facility at KATH, having completed the installation of a SPECT/CT [34]. As an alternative to these advanced imaging modalities, abdominal ultrasound and x‐ray are more commonly available and thus utilized for evaluation of distant metastases, but with more limited sensitivity [21].

### 3.2. Treatment

#### 3.2.1. Chemotherapy

Of the 16 regions in Ghana, 50% do not currently offer chemotherapy treatment [35]. Eighteen hospitals across Ghana offer one or more standard forms of combination chemotherapy [35], including the KATH, the CCTH, and the Korle Bu Teaching Hospital, all located in major urban centers. Studies conducted at these hospitals support that the vast majority of patients present with advanced disease, with 61.4% of patients with Stage III or IV disease at the KATH [36] and 78.8% of patients with Stage III or IV disease at the CCTH [23]. Whereas it is more common for women across the globe to undergo chemotherapy due to differences in stage at presentation, it is relatively more typical for women in Ghana to receive neoadjuvant chemotherapy, as demonstrated by 41% of breast cancer cases that received chemotherapy in the neoadjuvant setting at the Korle Bu Teaching Hospital in Ghana from 2005 to 2014 [37]. Similarly, of breast cancer cases seen at the KATH from 2008 to 2011, 33.5% received no treatment, 31.2% received neoadjuvant chemotherapy, 19.4% received adjuvant chemotherapy, 13.2% received palliative treatment, and 2.7% received “other treatment” [36]. Similarly, at CCTH, among surgical patients between 2011 and 2019, 64.5% received neoadjuvant treatment, and 32.6% received adjuvant treatment [23]. These data reflect the greater prevalence of advanced‐stage diagnoses and the need to downstage tumors with neoadjuvant chemotherapy to improve surgical resectability.

#### 3.2.2. Endocrine and Targeted Therapy

Sixteen hospitals across Ghana offer endocrine therapy, with 15 offering tamoxifen with or without other agents and one offering only aromatase inhibitors [35]. The hospitals that offer tamoxifen are located in the Ashanti, Bono, Central, Eastern, Greater Accra, Northern, Volta, and Western regions. Aromatase inhibitors are available in 14 hospitals in Ghana in the Ashanti, Bono, Central, Eastern, Greater Accra, Volta, and Western regions. Additionally, anti‐HER2 targeted therapy is available in six hospitals in Ghana that span the Ashanti, Central, Greater Accra, and Volta regions [35]. Since July 2019, trastuzumab has been covered by the NHIS for Ghanaian patients with HER2‐positive breast cancer, making Ghana the first country in sub‐Saharan Africa to include the drug on its national formulary [38]. Coverage encompasses two indications: early stage HER2‐positive breast cancer, where trastuzumab is administered for 1 year in the adjuvant setting following surgery and/or chemotherapy to reduce recurrence risk, and metastatic HER2‐positive breast cancer, where coverage similarly extends for 1 year under the NHIS. As the NHIS specifically covers the subcutaneous formulation, administered every 3 weeks, 1 year of treatment corresponds to approximately 17–18 cycles [38]. For women with hormone‐positive cancers that need ovarian function suppression, the cost of medications can be prohibitive, leaving oophorectomy as the alternative.

#### 3.2.3. Alternative Therapy

Alternative therapies are utilized in Ghana. One study provided evidence that, before seeking medical care, women believing the undiagnosed breast cancer to be a boil were shown to seek herbal medicine [36]. Women who presented with pain as the initial symptom of their breast cancer diagnosis were found to use over‐the‐counter pain medications or faith healing to manage symptoms rather than oncologic treatments [36]. Many women found alternative therapy providers to be more accessible as they are more often consulted for common ailments and are available within their local communities [36]. Further, alternative therapy providers are often viewed as more trusted in Ghana than orthodox medicine providers, especially if the patient views the disease′s cause to be associated with spiritual powers [39]. Of breast cancer patients seen at the KATH from 2008 to 2011, 11.3% sought alternative therapies of herbal medicine [36].

#### 3.2.4. Surgical Treatment

Depending on the extent of disease, geospatial availability, patient preferences, and finances, surgical options for breast cancer treatment include breast‐conserving surgery (BCS) or mastectomy, with or without reconstruction. According to the National Comprehensive Cancer Network (NCCN), basic surgery is defined as the minimum care to improve disease outcomes and includes mastectomy with axillary lymph node dissection (ALND) [40]. As of 2023, across nine regions, 33 hospitals offered basic surgery for breast cancer [35]. Thirty‐two of those 33 hospitals also offered BCS, whereas 10 of 33 offered sentinel lymph node biopsy. Nine of the 310 total surveyed hospitals offered options for reconstruction. Forty‐five additional hospitals offered mastectomy or lumpectomy but did not provide any axillary management and, therefore, are excluded from the basic care definition. Only three hospitals offered all services of sentinel lymph node biopsy and reconstruction in addition to basic care, two of which were located in Greater Accra and the other in the Ashanti Region [35]. Although mastectomy and ALND are more commonly performed due to the extent of disease, these figures highlight the regional discrepancies in access to comprehensive surgical management of breast cancer.

Where reconstruction is offered, Ghanaian centers have relied primarily on autologous flap techniques rather than implant‐based approaches, reflecting local cost and resource constraints. At KATH, a structured breast reconstruction program evaluated the latissimus dorsi musculocutaneous flap alongside lower abdominal and midabdominal transverse rectus abdominis myocutaneous (TRAM) flap techniques, ultimately identifying the midabdominal TRAM flap as the most reliable, well‐perfused, single‐stage option achievable within local resource constraints [41]. This reliance on autologous reconstruction rather than implant‐based reconstruction, which requires sustained access to prosthetic devices and specialized monitoring, is consistent with the limited number of centers nationally equipped to offer any reconstructive option. This underscores the importance of expanding trained plastic and reconstructive surgery capacity to broaden access beyond the few hospitals currently able to provide it.

Stigma and fear of mastectomy are commonly cited as a major driver of late presentation, which has been associated with lower functional well‐being and body image than patients who underwent BCS or mastectomy with reconstruction [42]. However, knowledge of surgical options is limited, as reported by a survey of 636 breast cancer participants, where only 7% of patients were aware that BCS is a potential treatment option for breast cancer. Patients diagnosed at earlier stages exhibit greater awareness of treatment options compared with those diagnosed at advanced stages, which may reflect that early stage patients have had more treatment options offered to them during shared decision‐making [43].

It is notable that patients at late stage, though less likely to learn about BCS as a treatment option, are more likely to receive neoadjuvant chemotherapy to improve resectability before surgery [37]. It is hypothesized that this neoadjuvant therapy administered to enhance surgical outcomes often does not reduce the cancer to an extent that would have expanded treatment options to BCS if the tumor was diagnosed in an earlier stage.

#### 3.2.5. Radiation Therapy

Radiation treatment for breast cancer, including whole breast, chest wall, and nodal irradiation, is an important treatment modality for women with advanced disease and those undergoing BCS. Though the standard treatment algorithm for BCS includes adjuvant radiation to the breast and potentially regional lymph nodes [44], radiation is only available at three hospitals throughout Ghana, two in the Greater Accra Region (one in the public and the other in the private sector), and one within the Ashanti Region (public sector) [35], making access to radiation a significant barrier to comprehensive breast cancer treatment for patients throughout much of the country.

Travel to and from radiation centers for weeks‐long treatment poses a significant barrier for patients in less resourced regions in Ghana. Advances in hypofractionation may alleviate the time toxicity associated with lengthy radiation treatments. Hypofractionation delivers a higher dose of radiation over a shorter treatment course, reducing time toxicity [45]. Currently, the National Centre for Radiotherapy Oncology and Nuclear Medicine, Korle Bu Teaching Hospital, offers hypofractionated treatment, which delivers 40.0005 Gy over 3 weeks compared with the conventional 50 Gy over 5 weeks. Studies comparing hypofractionation to conventional radiation therapy show a decrease in acute radiation‐induced toxicity [46]. The center provides many of the advanced radiation services to patients in neighboring areas around Accra.

In terms of coverage for radiotherapy, the NHIS reimburses breast and cervical cancer patients for a fixed number of sessions [47]. Although breast radiotherapy is included in the official NHIS benefits package, many patients report high out‐of‐pocket expenses if care requires additional treatments [47]. Moreover, some hospitals report not receiving full reimbursement from NHIS, contributing to additional out‐of‐pocket costs to patients [47].

#### 3.2.6. Treatment Outcomes

The pattern of treatment and outcomes in Ghana reflects the high burden of advanced‐stage disease at presentation. Recurrence and metastasis data illustrate the downstream clinical burden: A retrospective study at KATH found that 47.6% of patients developed metastases to distant sites, most commonly the lungs, bones, liver, and brain, whereas locoregional relapse remained comparatively low at 3.4%—suggesting that when treatment is accessed early, initial disease control can be achieved [48]. Molecular subtype plays a significant role in these outcomes. The basal/triple‐negative subtype, which accounts for over 40% of breast cancer cases at KATH, carries the highest risk of distant metastasis, with approximately 35% of patients with TNBC experiencing recurrence—often at multiple organ sites with limited benefit from additional therapies [48]. These patterns underscore the need for earlier diagnosis, equitable access to adjuvant chemotherapy, and treatment protocols tailored to the molecular profile of breast cancer in Ghana.

#### 3.2.7. Posttreatment Surveillance

Following active treatment, structured surveillance is critical for early detection of recurrence and ongoing monitoring of treatment‐related complications. In Ghana, posttreatment surveillance remains largely opportunistic rather than protocol‐driven. Many women with breast cancer tend to re‐engage with the health system only when new symptoms arise, reflecting a reactive model of follow‐up care that limits opportunities for early identification of recurrence [39]. Limited collection of demographic and contact information further hinders continuity of care, pointing to the value of strengthening systems for patient tracking and recall [49].

Routine surveillance modalities—including lymph node exams, axillary ultrasounds, and breast imaging via mammography or sonography—are not yet widely available outside major urban centers. The NHIS does not currently cover breast cancer–specific surveillance, with only routine noncancer‐specific follow‐up visits included under the scheme [50]. Geographic concentration of imaging and follow‐up services in Greater Accra and the Ashanti regions further limits access for patients in peripheral areas, for whom surveillance may require long‐distance travel and repeated visits [20].

Strengthening posttreatment surveillance through expanded imaging access, standardized follow‐up protocols, and NHIS coverage of breast cancer–specific monitoring represents a critical opportunity to improve long‐term outcomes. With greater investment in access and public awareness, the current reactive model could be replaced with proactive, protocol‐driven surveillance embedded in the posttreatment journey.

#### 3.2.8. Palliative Care Services

Palliative care in Ghana remains nascent, with dedicated services anchored at tertiary institutions and significant opportunities to expand access and awareness. Reflecting this, a retrospective study of 154 patients with metastatic breast cancer at Korle Bu Teaching Hospital found that 85.1% were managed with palliative intent, underscoring the system′s commitment to dignified care even when curative options are not available [51]. Yet, in practice, this commitment has not yet reached most patients. A cross‐sectional study of 176 women with advanced breast cancer found that whereas an outpatient palliative care unit existed at KATH, 84.7% of participants had not yet accessed it, and most specialized services—including physiotherapy, psychology, social work, and transport assistance—were not yet widely available [52]. Financial constraints, geographic distance, and limited awareness of services were the primary barriers to access—factors that are amenable to targeted intervention [52].

What the literature further reveals, across provider and patient perspectives alike, is a picture of untapped potential. Health care providers articulated a nuanced understanding of the multidimensional needs women with advanced breast cancer carry—spanning informational, psychological, physical, practical, social, and spiritual domains—reflecting the knowledge base already present to build more integrated care models [53, 54]. Women with breast cancer in Kumasi echoed this, expressing a desire for more personalized guidance and clearer referral pathways, suggesting that even modest improvements in navigation could meaningfully shift the patient experience [54]. Nurses bring considerable frontline expertise in pain management, home care, and psychosocial support and point to specialist training and resource investment as the areas that would most expand what they can offer [55]. Strengthening this workforce is therefore not a distant aspiration but a concrete, near‐term lever—and early evidence already supports its promise: A pilot nurse‐led program in the Volta Region demonstrated significant improvements in symptom distress, pain severity, and quality of life for women receiving palliative chemotherapy, showing that structured models can succeed in the Ghanaian context [56]. With greater NHIS coverage of supportive services, integration into existing oncology pathways, and investment in specialist training, expanding palliative care access in Ghana is both achievable and urgent.

### 3.3. Survivorship

Per NCCN guidelines, cancer survivorship care encompasses the physical, psychosocial, and functional consequences of cancer and its treatment—beginning at diagnosis and continuing throughout life. In Ghana, survivorship care is an evolving area of focus as emerging evidence and community‐driven initiatives are beginning to define what a more comprehensive model could look like [57]. While these clinical challenges are formidable, they are compounded by the lack of structured follow‐up care. Although many women currently face unclear next steps after treatment, efforts to expand access to surveillance, symptom management, and psychosocial support offer promising opportunities for improvement. In this context, survivorship must be understood not just as a clinical phase but as a multidimensional experience that requires long‐term investment in medical, emotional, psychosocial, and logistical support. Survivorship research has often been underrepresented in the broader landscape of cancer care studies, with most efforts to date focusing on treatment delivery and outcomes. In Ghana, emerging insights from these treatment‐focused studies are beginning to shed light on the gaps in posttreatment care and can be utilized to inform future priorities. Physical therapy treatment for lymphedema is not currently standard in Ghana. The current understanding of lymphedema and how to treat it is due to the occurrence of lymphatic filariasis, a parasitic disease transmitted by mosquitoes [58]. Notably, ALND has been identified as a statistically significant risk factor for lymphedema [59], which is associated with substantial long‐term morbidity, including recurrent infections, functional impairment, reduced quality of life, and increased mortality in advanced or untreated cases [60]. Addressing lymphedema therefore represents a critical opportunity to strengthen survivorship care as part of high‐quality, comprehensive breast cancer treatment in Ghana. As awareness around survivorship continues to grow, these insights provide a foundation for developing more connected and supportive posttreatment care pathways.

While breast reconstruction can contribute to improved self‐confidence and self‐image in women undergoing breast surgery, it is not the current standard of care for breast cancer patients in Ghana. Of women who declined breast reconstructive surgery as a part of their surgical treatment, influences on such decisions included the cost, the feeling that they did not need it, or the worry that it would affect treatment [61]. Additional information regarding the availability of postmastectomy garments, external breast prostheses, and lymphedema compression garments is available at breast clinics and often via regular donations from breast cancer survivor organizations or is available for out‐of‐pocket purchases in larger cities.

Despite the current absence of formal national programming for survivors, community‐based efforts have demonstrated what is possible. The Peace and Love Survivors Association (PALSA) emerged as a grassroots initiative with a vision to “create a community of breast cancer survivors reintegrating into society emotionally, physically, [and] psychologically healed/content and by so doing demystify breast cancer in Ghana” [62]. Whereas the present status of the organization is unclear, PALSA was active as recently as February 2024, offering free surgical services through its affiliated hospital and maintaining engagement via social media [62]. Initiatives like PALSA represent an important foundation for future survivorship efforts—offering a model that is community‐driven, culturally grounded, and survivor‐centered.

### 3.4. Financing Care

The NHIS was implemented in 2003 to reduce out‐of‐pocket health expenditures and improve equity in access to health care across Ghana. Membership in the NHIS is legally mandated under national health insurance legislation, including provisions articulated in the National Health Insurance Regulations (Act 652), whereas the scheme is currently governed by the National Health Insurance Act of 2012 (Act 852). Since its implementation, the NHIS has substantially expanded insurance coverage nationwide and remains the primary payer of health services in Ghana. Whereas the implementation of the NHIS has spurred a large increase in the number of insured members of the population and provided access to important services, many services that are vital to breast cancer diagnosis, treatment, and surveillance are still not adequately covered, including CBE, routine mammography, and ultrasound services for screening. While basic chemotherapy and surgical services are covered by the NHIS, these services are available only at major hospitals; radiotherapy and targeted therapies are not fully covered, and there is nonexistent coverage of immunotherapies. In terms of follow‐up care and surveillance, only routine, non–breast cancer–specific appointments are covered for survivors [50].

According to the 2022 GDHS, health insurance coverage in Ghana is high, with 90.1% of women and 73.4% of men reporting health insurance enrollment [6]. Coverage is almost entirely provided through the NHIS, which accounts for more than 99% of reported insurance among insured individuals [6]. These figures reflect substantial expansion of insurance enrollment nationwide and align with the legal mandate for NHIS participation.

Despite widespread insurance coverage, health care utilization remains limited. Only 32% of women and 20% of men reported seeing a health care practitioner in the 6 months preceding the survey [6]. These findings demonstrate a substantial gap between insurance enrollment and actual use of health services.

Financial barriers appear to be a central contributor to this gap. The GDHS reports that insured women and men frequently incurred out‐of‐pocket payments for health services [6]. Diagnostic services were the most common source of these payments. More than half of insured women (53%) and nearly two‐thirds of insured men (65%) paid in full for diagnostic care despite having insurance coverage [6]. Insured women reported having paid in full for surgical care; insured men reported having paid for medicines and consultations [6]. These patterns indicate that insurance coverage does not consistently protect individuals from direct health care costs.

A lack of public awareness of the disease and its course leads to many women not knowing that chemotherapy requires multiple rounds of costly therapies, let alone the direct nonmedical and indirect costs associated with breast cancer treatment [63]. Direct costs of care include medical expenses and transportation, whereas indirect costs involve patient travel time, time away from productive ventures, caretaker time spent on care and travel, patient waiting time, and caretaker waiting time. For breast cancer treatment in Ghana, direct costs of care reflect 61.9% of total finances, whereas the remaining 38.1% encompasses indirect costs [63]. The cost of breast cancer surgery accounts for approximately 23% of total medical costs, with 6% due to chemotherapy, 9% due to prescriptions, and 16% due to imaging [64].

Within 1 year of active treatment, breast cancer patients in Ghana in 2019 paid a mean amount of 31,021.00 Ghana cedis (GHC) (≈$5500.20 USD). Cost varied significantly by facility type: The median cost at public facilities was 29,606.30 GHC (≈$5249.30 USD), whereas at private facilities, it was nearly double at 55,071.20 (≈$9744.40 USD) [63]. When compared with income levels in Ghana, these treatment costs are staggering. For context, the average monthly salary for Ghanaians ranges from 2344 GHC (≈$211.36 USD) for those with no formal education, 2500 GHC (≈$225.43 USD) with basic education, and 3704 GHC (≈$333.99 USD) with a secondary certificate. Only 6.0% of women and 9.5% of men attain higher education than a secondary certificate, and Ghanaians report an average monthly income of 4912 GHC (≈$442.92 USD) with a diploma and 10,404 GHC (≈$938.14 USD) with a degree or higher [6, 65]. For individuals without formal education, the average cost of treatment at a public hospital exceeds one full year′s salary, and the treatment at a private facility can cost more than 2 years of income. Even for the rare minority of women who are degree holders, private treatment can still amount to nearly half a year′s salary.

### 3.5. Transportation

#### 3.5.1. Finances of Transportation

The financial burden of transportation is one of the most frequently cited reasons for delay in initial diagnosis. Patients must often make multiple trips for consultation, biopsy, and imaging—sometimes at different facilities—and when facilities are distant from their home, this can pose a significant barrier to accessing the spectrum of care needed to diagnose and treat breast cancer [66]. As a result, some patients forego or interrupt treatment due to affordability, even when clinical services are available [67].

#### 3.5.2. Geographic Accessibility

Geographic accessibility is a key determinant of timely breast cancer screening and diagnosis in Ghana. Women in urban areas are more likely to engage in self‐breast and clinical examinations [14]. Currently, 41.3% of women live within 1 h of a mammography facility, and approximately 75% of the population is within 1 h of a facility offering pathology services [14, 20]. These facilities are largely concentrated in Greater Accra and the Ashanti regions (Figure 2C), contributing to longer intervals between symptom onset, diagnosis, and treatment in other parts of the country. Whereas chemotherapy and radiation services remain concentrated in a few urban hubs such as Accra, Kumasi, and Tamale, surgical services are somewhat more accessible, with hospitals offering breast cancer surgery present in at least nine of Ghana′s regions [68]. For patients outside these areas, care may require long‐distance travel, repeat visits, and overnight stays—factors that contribute to treatment delays, discontinuation of treatment, and poorer outcomes. Services such as endocrine therapy, surveillance imaging, lab work, and other interdisciplinary support are concentrated in urban referral hospitals, making follow‐up care particularly difficult for rural patients [20]. Despite these challenges, these data provide valuable insight into areas where access is growing and where continued progress can be made.

#### 3.5.3. Transportation Challenges and Solutions

Transportation delays are a key factor in Ghana′s health care access disparities. Studies have linked long travel times with late‐stage disease presentation, missed screenings, and treatment discontinuation [69]. These challenges disproportionately affect women, rural residents, and low‐income communities, further exacerbating existing inequities [66, 67]. Investments in road infrastructure, expansion of community‐centered models, and adoption of technologies like drone delivery and motorcycle ambulances are helping to close the access gap for not only breast cancer care but also all health care. Programs such as Moving Health demonstrate how locally tailored solutions can improve emergency response and routine care delivery [70]. Policy interventions are also essential. Mapping regional gaps in access, strengthening regional cancer registries, and integrating transportation needs into cancer control planning will be critical for building a more equitable system. By prioritizing transportation as a health determinant, Ghana can move closer to ensuring timely, affordable, and consistent care for all individuals affected by breast cancer.

### 3.6. Sociocultural Factors

In Ghana, women′s beliefs about breast cancer are shaped by rich and diverse cultural, spiritual, and religious frameworks that offer important context for understanding their health behaviors. Women often interpret breast cancer, among other illnesses, as a spiritual or ancestral message, reinforcing the interconnectedness of health, family, and tradition. For example, in Tamale, women commonly attributed breast cancer to curses, ancestral punishment, or divine consequences for perceived moral failings [71]. In Kumasi, some women believed breast cancer could result from spiritual causes such as punishment for not bearing children, or through supernatural forces [72]. These interpretations reflect deeply rooted cultural worldviews and offer meaningful insight into how health is contextualized and addressed within communities.

Religious and spiritual beliefs also play a meaningful role in coping strategies and treatment decisions. In Greater Accra, Tamale, and Kumasi, breast cancer was described in some cases as a test of faith or a spiritual condition—shaping decisions to pursue healing through prayer or faith‐based interventions [72]. Women expressed strong faith in spiritual healing, often turning to prayer camps or traditional remedies before accessing formal care. A metasynthesis of spiritual care provision for patients with advanced cancer across sub‐Saharan Africa similarly found that patients routinely combined orthodox treatment with spiritual and religious healing and expressed a desire for such care to be formally integrated into oncology care pathways [73]. However, there is still a notable blend of perspectives, with some women embracing both medical and spiritual care [72]. This represents a promising space for developing integrative care models that align with patients′ values while supporting timely diagnosis.

Addressing breast cancer also involves confronting common myths and misconceptions about its causes and risk factors. In a study of university students in Accra, 43.4% believed breast cancer could be caused by a man sucking a woman′s breast, and 68% believed younger women were unlikely to develop the disease, despite global data showing rising incidence in young adults [72]. Further, other opinions sourced the cause of breast cancer to coins in bras, spider bites, or trauma [72]. While these beliefs are not medically supported, they reflect a desire to make sense of disease using familiar concepts. In the Volta Region, 46.1% of rural women recognized breast cancer as the most common cancer among women, though many held mixed views about its curability [74]. In Asokore, 54.6% believed breast cancer was not curable, and 19.6% were uncertain, suggesting the importance of addressing medical uncertainty with clarity and empathy. Cultural preferences regarding modesty also influenced screening access, with some women avoiding CBEs if they were performed by male providers [72].

Perceptions of breast cancer prognosis deeply influence how women interpret their future with the disease. One study found that breast cancer was viewed as rapidly fatal or even contagious, contributing to fear and, in some cases, social distancing from affected individuals [75]. Research in northern Ghana similarly found that the psychological burden of cancer was compounded by shame and stigma, with financial hardship driving social withdrawal and deepening the isolation of affected women within their communities [76]. These perceptions may delay patient presentation to the hospital or discourage open discussion of symptoms. It is worth acknowledging that these fears are not entirely unfounded: In Ghana, nearly half of patients present with metastatic disease at diagnosis, and the proportion of women diagnosed with distant metastatic breast cancer in sub‐Saharan Africa (5.6%–30.6%) is substantially higher than in North America (0%–6%), meaning that community perceptions of breast cancer as deadly may reflect lived experience rather than misconception [48, 77]. These concerns are further borne out in survival data: A pooled meta‐analysis of six Ghanaian breast cancer cohorts estimated a median overall survival of just 4.4 years and a 5‐year survival rate of only 46.5%, reinforcing that fear surrounding a breast cancer diagnosis in Ghana is rooted in real mortality risk rather than misconception alone [78]. At the same time, many women expressed hope and belief in recovery, especially when accurate information was provided or when care was delivered with cultural sensitivity [75]. Education that affirms women′s strength and resilience while correcting misconceptions about outcomes may be particularly effective in improving early detection and treatment.

### 3.7. Emerging Fields of Breast Cancer Care

#### 3.7.1. Genetic Testing

One‐third to more than one‐half of sub‐Saharan African breast cancer patients have TNBC [26], with BRCA1 germline mutations associated with increased rates of TNBC [79]. Within a population of breast cancer patients with a diagnosis of locally advanced disease and a positive family history to an unspecified degree at KATH from 2009 to 2011, surgical resection specimens were used to obtain fixed paraffin‐embedded samples to extract DNA and genotype for three different known pathogenic BRCA mutations including BRCA1 185delAG, BRCA1 5382insC, and BRCA2 6174delT, showing 61.7% of cases with at least one of these mutations, and 60.6% of TNBC subtype [79]. A prior study showed that Ghanaian women have approximately two times the frequency of pathogenic variants in BRCA1 and BRCA2 compared with women of European or Asian ancestry [80]. Notably, because the prevalence of BRCA mutations and TNBC is higher in women in Ghana, testing for genetic variants and population‐based education in genetics in breast cancer are of the utmost importance. This also has implications for additional systemic therapy with PARP inhibitors and cascade testing of family members who may harbor this high‐risk genetic variant. Whereas various programs throughout Ghana, including the UM‐KATH partnership, have displayed promising progress in testing for HR status to quantify TNBC, substantial progress must still be made in regard to genetic testing for BRCA genes in Ghana to make an impact on breast cancer preventative care [29].

Despite this need, BRCA 1/2 testing is not currently available as part of routine clinical care in Ghana; access to date has depended on international research collaborations, such as the UM‐KATH partnership, which has extracted DNA from archived paraffin‐embedded tumor tissue to genotype Ghanaian patients for BRCA1 mutations [29, 79]. Formal genetic counseling services are similarly lacking, with no dedicated genetic counseling workforce currently known to serve oncology patients in the country. This reflects a broader regional shortage; even in South Africa, with the continent′s most established clinical genetic infrastructure, a 2024 national audit identified only 13 practicing medical geneticists and 28 genetic counselors nationwide, representing just 10% and 5% of estimated capacity targets, respectively [81]. Without accessible testing and counseling pathways, Ghanaian patients and at‐risk family members currently have limited ability to benefit from cascade testing, risk‐reducing interventions, or genetically informed treatment selections such as PARP inhibitor therapy.

#### 3.7.2. Oncofertility

Oncofertility refers to the integration of reproductive medicine and oncology to preserve fertility for patients facing gonadotoxic cancer treatment. According to the NCCN Survivorship Guidelines, the opportunity to discuss fertility preservation—including oocyte, embryo, or ovarian tissue cryopreservation for women and sperm banking for men—is essential for all patients of reproductive age with a newly diagnosed cancer, before treatment initiation, regardless of cancer subtype or genetic mutation status [27, 82]. This is especially relevant in Ghana, where breast cancer is diagnosed at a markedly younger age than in high‐income settings: The mean age at diagnosis across sub‐Saharan Africa is 45–52 years, compared with approximately 62 years in the United States and Western Europe, and Ghanaian cohort data show that 13%–22% of cases occur in women aged 35 or younger. Whereas 100% of specialists in Ghana report awareness that cancer treatments impair fertility, only 32% of patients have a fertility discussion with their provider due to fertility wishes [83]. Yet childbearing intentions are generally strong in Ghana, and fear of infertility is commonly cited as a reason for noncompliance with breast cancer treatment [84, 85]. This gap is echoed in a recent multicenter study of young Ghanaian breast cancer patients, which found fertility preservation awareness of only about 39%, with cost, fear, and partner objection as the leading barriers to uptake [86]. These barriers are compounded by cultural and religious context: Qualitative work among Ghanaian women living with breast cancer further reveals that assisted reproductive technologies are widely perceived as unnatural and in tension with religious or cultural beliefs, framing conception as a sacred act, such that women who pursue fertility preservation may risk stigma or condemnation from family, community, or religious leaders—a perception reinforced by how few known success stories or testimonials patients have to draw on [87]. Cost, in particular, remains a decisive obstacle: IVF in Ghana typically costs between $3000 and $6500 per cycle, exceeding the country′s GNI per capita of approximately $2320, and the NHIS does not cover fertility treatment [88, 89]. It is notable that Ghana does have several facilities with infertility services, including in vitro fertilization [90–92]. In total, there are 22 assisted reproductive therapy centers which are concentrated in three regions across Ghana: Takoradi, Kumasi, and Accra [93]. Bridging the gap between fertility service availability and their integration into cancer care—through provider training, patient education, and policy support—is essential to optimize oncofertility service utilization and improve long‐term quality of life for young breast cancer patients in Ghana.

#### 3.7.3. Clinical Trials

In Ghana, all clinical trials are subject to mandatory registration with the Pan African Clinical Trials Registry and require regulatory clearance from the Food and Drugs Authority [94]. Between 2004 and 2024, 546 clinical trials were registered in Ghana. Of these, only three specifically investigated breast cancer, and they varied substantially in design and purpose. One was a breast cancer screening feasibility study conducted in collaboration with Pennsylvania State University [95]. A second was a multicountry interventional therapeutic trial sponsored by the International Atomic Energy Agency that evaluated a resource‐sparing postmastectomy radiotherapy regimen in patients with breast cancer in limited resource settings, including Ghana [96]. The third was a noninterventional epidemiological study sponsored by the US National Cancer Institute that investigated breast cancer risk factors among women in Ghana [97]. All three breast cancer trials enrolled only female participants, underscoring the ongoing need to include men in breast cancer research, particularly in studies exploring awareness, screening behavior, and genetic risk [95, 98]. These initiatives reflect growing momentum in Ghana′s clinical research landscape, but cancer‐specific trials, particularly for breast cancer, remain limited in both scope and number, underscoring the need for expanded research investment.

## 4. Discussion

Breast cancer is the most prevalent malignancy in Ghana. Furthermore, women are diagnosed at a younger age and with more advanced disease [1]. Optimizing care at each stage along the continuum—from preventative care to treatment to survivorship, along with optimizing the integrated variables of transportation and cost—is essential to improving the landscape of breast cancer in Ghana. Breast cancer screening services with imaging are primarily concentrated in urban centers. A critical shortage of trained pathologists and pathology services throughout the country represents a significant bottleneck to diagnosis and subtyping breast cancer. Medical treatment is largely palliative or offered in the neoadjuvant setting due to advanced disease; radiation therapy is not merely underutilized but is scarcely available. Basic surgical care for breast cancer is limited to 33 hospitals throughout the country. Financing treatment proves to be an incredible burden, and although the vast majority of Ghanaian citizens have insurance, it does not cover the costs of breast cancer screening and comprehensive treatment. These findings are consistent with broader evidence demonstrating that higher Universal Health Coverage Service Coverage Index levels are independently associated with lower breast cancer age‐standardized mortality rates, reinforcing the urgency of closing coverage gaps for screening and treatment in Ghana to advance equitable breast cancer outcomes [99]. Access to care is limited by transportation to specialized centers where services are offered, emphasizing the need to improve access to regional care. Community education efforts are important to address sociocultural beliefs surrounding the causes and treatment options of breast cancer. Moreover, though efforts are new and of a smaller scale, Ghana has continued to show promise in emerging fields of breast cancer care in relation to genetic counseling and testing, oncofertility, and clinical trials—all fields that influence the quality of life and outcomes of breast cancer patients in the country. Critical gaps in access to and quality of care between urban and rural populations underscore the need to strengthen rural health care infrastructure, expand the oncology workforce, and invest in decentralized service delivery to ensure equitable access to breast cancer care across the country.

There were several limitations present in conducting this comprehensive review of breast cancer care in Ghana. The absence of a national cancer registry in Ghana contributes to challenges in accurately assessing the current burden and trends of breast cancer incidence, treatment, and outcomes across the country. The secondary analysis in this study of the Ghana Health and Demographic Survey introduces several limitations, including that the women′s questionnaire samples only women aged 15–49, which they consider to be the standard reproductive age sampling frame. Women older than 49, who fall within the age range for which mammography screening is most strongly recommended, were not surveyed. As a result, the screening rates reported reflect uptake among women of reproductive age broadly rather than a clinically screening‐eligible population and likely underestimate true screening behavior among older, higher risk women. Additionally, this review is limited in its representation of male breast cancer. The men′s questionnaire of the Ghana Health and Demographic Survey did not collect equivalent data about breast cancer, precluding sex‐disaggregated analysis and furthering the discrepancy of male representation in breast cancer literature. Inconsistencies across studies—such as variation in methodologies and reporting standards—further complicate efforts to synthesize conclusions. The inclusion of community organization websites allows for a more all‐encompassing view of the landscape of breast cancer beyond peer‐reviewed publications but introduces possible information bias, as our search was limited to organizations with an Internet presence. The accuracy of heatmap data is limited by the fact that imaging and pathology data were drawn from cross‐sectional surveys conducted in 2019–2021, whereas the population data for each region was collected in 2021. These calculations assume regional populations have remained stable.

Further research is needed to better understand how systemic factors such as insurance enrollment, out‐of‐pocket costs, diagnostic centralization, and transportation influence breast cancer outcomes in Ghana. Identifying where and how patients encounter delays can inform policies that promote earlier detection and more comprehensive care. Strengthening regional cancer registries, improving data capture on treatment and survivorship, and mapping geographic gaps in access are critical for building a more comprehensive cancer control strategy. As Ghana expands its diagnostic and pathology infrastructure, future studies should evaluate how decentralization affects timeliness of care, treatment continuity, and regional equity in outcomes.

Equally important is research that centers the patient experience. Educational approaches that empower women while addressing common fears and misunderstandings about breast cancer may play a key role in improving early presentation and treatment adherence. Understanding how awareness of surgical options influences care‐seeking behavior is important, particularly when it comes to the availability and affordability of oncoplastic reconstruction. Insights from this could guide the development of more effective patient counseling and support services. Future work should consider the underrepresentation of men in breast cancer research—not only as patients but also as caregivers and community stakeholders—to ensure that educational efforts and support systems are inclusive and effective.

## 5. Conclusion

This review offers a timely contribution to the growing body of work on breast cancer care in Ghana. By examining national patterns in screening and diagnosis, treatment availability, and survivorship, it highlights both the progress already made and the opportunities that lie ahead. The findings reinforce the importance of expanding equitable access through data‐driven planning, patient‐centered education, and system‐wide coordination. With continued investment and collaboration across sectors, Ghana is well‐positioned to lead innovative, inclusive approaches to breast cancer care. This work provides a strong foundation for future research, policy action, and advocacy focused on building a more responsive and resilient breast cancer care system—one that meets patients where they are and supports them every step of the way.

## Author Contributions

Ms. Shannon C. Lyden and Ms. Emma Gabriella O′Connor contributed equally to the conceptualization, data curation, formal analysis, project administration, visualization, and the writing of the original draft of this manuscript. Ms. Katie Kenny contributed via software, creating the figures for the manuscript, as well as through data curation and writing of the original draft. Ms. Hannah Wang contributed via data curation and writing of the original draft. Ms. Katerina L. Aris, Ms. Caroline Lommer, and Ms. Jessica Keane all contributed via conceptualization, data curation, and writing—original draft. Dr. Ishmael Kyei, Dr. Anita Eseenam Agbeko, Dr. Abena Yeboah Aduse‐Poku, and Dr. Bridget Oppong contributed via writing—review and editing. Dr. Andrea N. Riner contributed via the supervision of this project as well as the conceptualization, methodology, project administration, and writing—review and editing. Shannon C. Lyden and Emma Gabriella O′Connor are considered co‐first authors.

## Funding

No funding was received for this manuscript.

## Conflicts of Interest

The authors declare no conflicts of interest.

## Supporting information


**Supporting Information** Additional supporting information can be found online in the Supporting Information section. File S1: Search terms by topic.

## Data Availability

The data that support the findings of this study are available from the corresponding author upon reasonable request.
