# Supplementary material for: Breast Cancer Care in Ghana: A Review of the Continuum of Care and Progress Toward Universal Health Coverage
Source: Int J Breast Cancer. 2026 Sep 16;2026:3787700. doi: 10.1155/ijbc/3787700 (PMC13583168; doi:10.1155/ijbc/3787700)
Supplement: Supplementary file 1 [file IJBC-2026-3787700-s001.docx]

**Appendix. Search Terms by Topic**

**Screening & Diagnosis**

Clinical breast exam Ghana; mammography Ghana; breast ultrasound Ghana; breast cancer screening Ghana; MammaCare Ghana; breast cancer pathology Ghana; fine needle aspiration breast Ghana; core needle biopsy Ghana; immunohistochemistry breast cancer Ghana; hormone receptor testing Ghana; HER2 testing Ghana; triple-negative breast cancer Ghana; FISH testing breast cancer Ghana; CT scanner availability Ghana; MRI breast cancer Ghana; PET-CT Ghana; bone scintigraphy Ghana; SPECT/CT Ghana

**Treatment**

Breast cancer treatment Ghana; chemotherapy Ghana; neoadjuvant chemotherapy Ghana; adjuvant chemotherapy breast cancer Ghana; endocrine therapy Ghana; tamoxifen Ghana; aromatase inhibitor Ghana; trastuzumab Ghana; HER2 targeted therapy Ghana; oophorectomy Ghana; herbal medicine breast cancer Ghana; alternative therapy cancer Ghana; faith healing cancer Ghana; mastectomy Ghana; breast-conserving surgery Ghana; axillary lymph node dissection Ghana; sentinel lymph node biopsy Ghana; breast reconstruction Ghana; radiation therapy breast cancer Ghana; radiotherapy across Ghana; hypofractionation Ghana

**Survivorship**

Breast cancer survivorship Ghana; cancer surveillance Ghana; axillary ultrasound surveillance Ghana; lymphedema Ghana; lymphatic filariasis Ghana; post-mastectomy garments Ghana; breast prosthesis Ghana; PALSA Ghana; breast cancer support group Ghana

**Financing Care**

National Health Insurance Scheme Ghana; breast cancer insurance coverage Ghana; out-of-pocket cost breast cancer care Ghana; cost of breast cancer treatment Ghana; direct and indirect costs cancer Ghana

**Transportation**

Transportation barriers cancer care Ghana; geographic accessibility breast cancer Ghana; rural healthcare access Ghana; drone delivery healthcare Ghana; motorcycle ambulance Ghana; Moving Health Ghana

**Sociocultural Factors**

Breast cancer beliefs Ghana; breast cancer stigma Ghana; breast cancer myths Ghana; spiritual beliefs illness Ghana; breast cancer awareness Ghana; breast cancer curability perceptions Ghana

**Emerging Fields**

BRCA1 mutation Ghana; BRCA testing Ghana; genetic counseling cancer Ghana; PARP inhibitor Ghana; oncofertility Ghana; fertility preservation cancer Ghana; in vitro fertilization Ghana; clinical trials breast cancer Ghana; Pan African Clinical Trials Registry

**Background/Additional Context**

Breast cancer Ghana; breast cancer epidemiology Ghana; breast cancer incidence Ghana; breast cancer mortality Ghana; cancer burden Ghana; cancer registry Ghana; cancer sub-Saharan Africa; breast cancer age Ghana; healthcare system Ghana; insurance Ghana; Ghana health regions; universal health coverage breast cancer
